# Characterization of Human Colon Organoids From Inflammatory Bowel Disease Patients

**DOI:** 10.3389/fcell.2020.00363

**Published:** 2020-06-04

**Authors:** Emilie d’Aldebert, Muriel Quaranta, Morgane Sébert, Delphine Bonnet, Sylvain Kirzin, Guillaume Portier, Jean-Pierre Duffas, Sophie Chabot, Philippe Lluel, Sophie Allart, Audrey Ferrand, Laurent Alric, Claire Racaud-Sultan, Emmanuel Mas, Céline Deraison, Nathalie Vergnolle

**Affiliations:** ^1^IRSD, INSERM, INRA, ENVT, UPS, Université de Toulouse, Toulouse, France; ^2^Department of Internal Medicine and Digestive Diseases, CHU Purpan, Toulouse, France; ^3^Pole Digestif, CHU de Toulouse, Toulouse, France; ^4^Urosphere SAS, Toulouse, France; ^5^Plateforme d’Imagerie, CPTP, INSERM, INRA, ENVT, UPS, Université de Toulouse, Toulouse, France; ^6^Unité de Gastroentérologie, Hépatologie, Nutrition, Diabétologie et Maladies Héréditaires du Métabolisme, Hôpital des Enfants, CHU de Toulouse, Toulouse, France; ^7^Department of Physiology and Pharmacology, University of Calgary, Calgary, AB, Canada

**Keywords:** inflammation, Crohn’s disease, ulcerative colitis, organoid, intestine

## Abstract

Inflammatory Bowel Diseases (IBD) are chronic inflammatory disorders, where epithelial defects drive, at least in part, some of the pathology. We reconstituted human intestinal epithelial organ, by using three-dimension culture of human colon organoids. Our aim was to characterize morphological and functional phenotypes of control (non-IBD) organoids, compared to inflamed organoids from IBD patients. The results generated describe the epithelial defects associated with IBD in primary organoid cultures, and evaluate the use of this model for pharmacological testing of anti-inflammatory approaches. Human colonic tissues were obtained from either surgical resections or biopsies, all harvested in non-inflammatory zones. Crypts were isolated from controls (non-IBD) and IBD patients and were cultured up to 12-days. Morphological (size, budding formation, polarization, luminal content), cell composition (proliferation, differentiation, immaturity markers expression), and functional (chemokine and tight junction protein expression) parameters were measured by immunohistochemistry, RT-qPCR or western-blot. The effects of inflammatory cocktail or anti-inflammatory treatments were studied in controls and IBD organoid cultures respectively. Organoid cultures from controls or IBD patients had the same cell composition after 10 to 12-days of culture, but IBD organoid cultures showed an inflammatory phenotype with decreased size and budding capacity, increased cell death, luminal debris, and inverted polarization. Tight junction proteins were also significantly decreased in IBD organoid cultures. Inflammatory cytokine cocktail reproduced this inflammatory phenotype in non-IBD organoids. Clinically used treatments (5-ASA, glucocorticoids, anti-TNF) reduced some, but not all parameters. Inflammatory phenotype is associated with IBD epithelium, and can be studied in organoid cultures. This model constitutes a reliable human pre-clinical model to investigate new strategies targeting epithelial repair.

## Introduction

Inflammatory Bowel Diseases (IBD) are chronic inflammatory disorders of the intestine that include Crohn’s disease (CD) and ulcerative colitis (UC). IBD cause lifelong inflammatory disorders and severely impair the quality of life in patients (Panaccione et al., 2005). Current treatments target the inflammatory reaction *per se*, and in particular the recruitment of inflammatory cells (Neurath, 2017). However, the idea that a healed intestinal epithelium is necessary in addition to inhibition of immune cell recruitment has emerged. Mucosal healing is now considered as the next therapeutic challenge for IBD. This means that not only symptomatic improvement is expected with treatment, but also endoscopic remission, showing repaired tissues (absence of frailty, blood, erosion or ulcers) (Dave and Loftus, 2012). Indeed, a more favorable prognosis is associated with mucosal healing: patients having lower relapse and hospitalization rates (Peyrin-Biroulet et al., 2011; Burisch et al., 2013). Some of the current treatments used in the clinic are considered to favor, at least in part epithelial healing. This is the case for Mesalamine (5-ASA) (Baumgart et al., 2005), but also for anti-Tumor Necrosis Factor (TNF) (Fischer et al., 2013) and glucocorticoid treatments, which restore barrier function (Fischer et al., 2014). However, their effects on epithelial functions are considered very limited and the hope for future treatments is to strengthen the effects of therapies on epithelial healing.

The consensus to explain IBD’s etiology is to consider that in genetically predisposed individuals, a combination of immune disorders with intrinsic (epithelial barrier function for instance) and extrinsic (microbiota) factors lead to the development of chronic inflammation. Genome-wide association studies have identified a number of epithelial mediators and epithelial signaling pathways associated with IBD (McCole, 2014). These studies brought the epithelium at the forefront for the development of new therapeutic strategies, although epithelial dysfunctions in IBD have not been exhaustively defined yet (Khor et al., 2011; Rivas et al., 2011; McCole, 2014). Novel models focusing on epithelial biology are therefore necessary in the context of IBD. Such models are important both to better characterize epithelial dysfunctions associated with IBD and to assess new therapeutic strategies aiming at promoting intestinal epithelium healing.

The technological breakthrough represented by the culture of organoids from isolated tissue stem cells now allows to re-create *in vitro* an intestinal epithelial organ (Sato and Clevers, 2013). This technology is based on the isolation of intestinal crypts, which are then cultured in three-dimensions. In the presence of appropriate growth factors, intestinal stem cells present in the isolated crypts proliferate and enter into differentiation processes, recreating a complex epithelium, which contains all cell types that compose the intestinal epithelium (paneth cells, enteroendocrine cells, goblet cells, enterocytes, tuft cells, etc.). The epithelium generated by three-dimension cultures of isolated crypts closes on itself, forming a sphere, in which epithelial cells are orientated with their apical side toward the lumen (Sébert et al., 2018). While a number of studies have employed culture organoids from intestinal crypts (Sugimoto et al., 2018; Yip et al., 2018; Ramesh et al., 2019), only very few studies have investigated the possibility to culture organoids from IBD patient-isolated intestinal crypts (Dotti et al., 2017; Noben et al., 2017; Howell et al., 2018). Importantly, they reported transcriptional or methylation differences between organoids from UC or CD patients compared to controls (Dotti et al., 2017; Howell et al., 2018). Both studies suggested that intestinal epithelial cells undergo changes during IBD development that could be involved in pathogenesis. However, none of these two studies has performed any characterization of the morphology, cell composition or functions of IBD organoid cultures. The potential for human IBD organoid cultures to be used as a model to test therapeutic options that could target the epithelium in IBD has not yet been addressed either. Here, we have characterized the morphological and functional phenotype of IBD patient’s epithelium by using organoid cultures. Further, we have tried to establish whether organoid cultures from IBD patients could be used to test therapeutic approaches on epithelial healing.

## Materials and Methods

### Human Tissue Materials

Biological samples were obtained from individuals treated at the Toulouse University Hospital who gave informed consent. The MICILIP research protocol was approved by the national ethics committee (#NCT01990716) and was financially supported by the Toulouse University Hospital (Denadai-Souza et al., 2018). The biocollection that included colonic resections was approved under the CODECOH national agreement: Colic collection: DC2015-2443). These samples were freshly collected from non-IBD controls (healthy zones of tissues resected from patients with colorectal cancer or endometriosis) and from IBD patients (in non-inflammatory zones). Tissues were collected from 26 patients with Crohn’s disease, 8 patients with ulcerative colitis, and from 18 non-IBD patients. Characteristics and treatments for patients are provided in Supplementary Table S1.

### Isolation and Culture of Colon Crypts

Fresh colonic tissues or biopsies were harvested and colon crypts were isolated and cultured mostly as previously described (Jung et al., 2011; Sato et al., 2011), with some adaptation that are described in Supplementary Material.

### Treatments

Control organoids were treated by a pro-inflammatory cytokines cocktail [IL-1β, IL6, TNF-α (PeproTech)] at the concentration of 10 ng/ml (Supplementary Figure S1). IBD organoids were treated independently by three routinely used medications for the treatment of IBD patients: (anti-TNFα or 100 μM), methylprednisolone (10 or 100 μM), and 5-ASA (50 or 500 μM) (Supplementary Figure S2).

### Immunofluorescence Labeling

Organoids (in chamber slide) were fixed with 4% formaldehyde (FA, Sigma Aldrich) in Hank’s Buffered Salt Solution (HBSS, Gibco) at 37°C for 40 min. Organoids were permeabilized with 0.5% triton X-100 (Sigma Aldrich) in HBSS at RT for 40 min and were incubated with a blocking buffer containing 3% bovine serum albumin (BSA, Sigma Aldrich) in HBSS overnight at 4°C. Organoids were incubated 2 h at 37°C with primary antibodies. Next, organoids were incubated with secondary antibodies 1.5 h at 37°C and then stained for 20 min with DAPI (Invitrogen) and Phalloidin (see Supplementary Table S2 for all antibodies) (Cenac et al., 2015). Finally, slides were mounted with Vectashield mounting medium (VectoLaboratories).

### Colonoid Imaging

The images of organoids were taken by confocal microscopy with a Leica SP8. Image J software was used to process and analyze images. Ten images at 20X and 10 images at 63X objectives were analyzed for each well, and an average of 2 wells were analyzed for each condition. To assess barrier function, organoids were incubated with 1 mg/ml of fluorescein isothiocyanate (FITC)-labeled dextran 4-kDa for 2-h at 37°C, and images were analyzed on a confocal microscope with a X63 lense.

### RNA and Protein Extraction

After an average of 10 days of culture, organoids (8–10 wells in 48 wells plates) were incubated in cell recovery solution (Corning) for 30 min at 4°C. After centrifugation, pellet-containing cells were resuspended in RP1 buffer (NucleoSpin^®^ RNA/Protein kit, Macherey Nagel). Total RNA and protein were extracted according to the manufacturer’s protocol.

### Reverse Transcription, and Real-Time Quantitative PCR Analysis

One μg of each purified RNA preparation was reverse-transcribed using Maxima First Strand cDNA Synthesis Kit (Fermentas Life sciences) and cDNAs were analyzed by qPCR using relevant primers as previously described (Rolland-Fourcade et al., 2017). qPCR analysis was performed on a LightCycler^®^ 480 Real-time PCR system device (Roche Applied Science) using the LightCycler^®^ 480 SYBR Green I Master (Roche Applied Science), according to the manufacturer’s instructions, and specific primers (Supplementary Table S3). All experiments included a standard curve and all samples were analyzed in duplicate and expressed as a relative amount (2–ΔCt). Relative expression of targeted genes was compared to GlycerAldehyde-3-Phosphate DesHydrogenase (GAPDH).

### Western Blot

Fifty microgram of total proteins were separated by 4–20% Mini-Protean Gel (Bio-Rad). Separated proteins were transferred onto a nitrocellulose membrane. Primary antibodies (Supplementary Table S2) as well as peroxidase conjugated secondary antibodies were used according to standard Western blot procedures. Peroxidase was detected by using the ECL^TM^ Prime Western Blotting system (GE Healthcare). Quantification of signals was done using Image Lab software (Bio-Rad).

### Metabolic Activity Assay

Organoids were plated in 96-wells opaque plates after 6 or 9 days of culture. CellTiter-Glo^®^ 3D reagent (Promega, France) was added to each well (volume 1:1), and incubated for 30-min at room temperature (Sébert et al., 2018). Triplicate measures were performed for each condition. Luminescence was measured with a Varioskan Flash (Thermo Scientific), and images of each well were taken with Apotome, in order to evaluate the surface occupied by all organoids (Fiji software). Results were normalized with the ATP standard curve and were expressed as ATP concentration per total surface of organoids.

### Statistical Analysis

Data are expressed as mean ± SEM. Analyses were performed using the GraphPad Prism 5 software. Statistical significance was determined by Student’s *t*-test, Mann–Whitney, and Wilcoxon tests where appropriate. Significance was accepted for *p* < 0.05.

## Results

### Characterization of Colonoid Cultures

**FIGURE 1:**
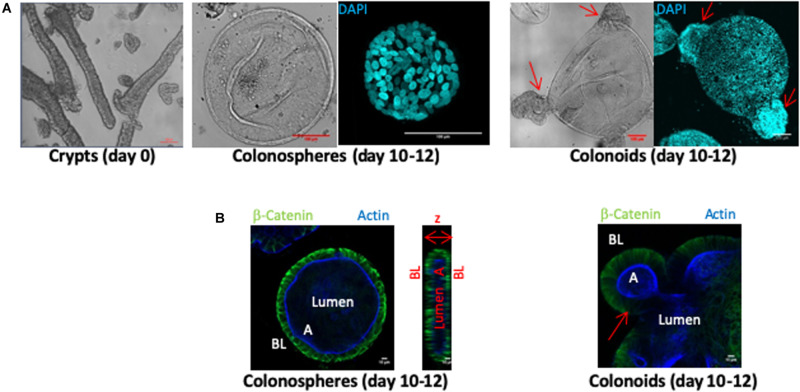
*Ex vivo* culture of human colon crypts from control patients. All images were acquired with SP8 confocal microscope at 20X and 63X objectives and analyzed by Image J software. **(A)** Isolated colon crypts at day 0 (left panel), and after 10–12 days of culture (middle and right panels) (scale bar 100 μm). Colored confocal pictures shows DAPI nuclei labeling (cyan) in a 3D reconstruction. Ten to twelve-days cultures showed either perfectly rounded structures called colonospheres (middle panel), or budding structures called colonoids (right panels). **(B)** β-catenin labeling (green) stained basolateral side (BL) and actin labeling (blue) stained apical side **(A)** of organoid epithelial cell monolayers, defining a central lumen, in cross section of colonospheres (left panel) and colonoids (right panel) (scale bar 10 μm). Arrows indicated buds.

Human colon crypts were purified from fresh tissue samples obtained from colectomies or biopsies harvested in healthy zones of non-IBD patient tissues. Crypts fragments (Figure 1A day 0 panel) were cultured in a three-dimensional Matrigel matrix for 10–12 days. CD45 immunostaining was negative in all crypt fragment samples, indicating that no inflammatory cells were present before the organoid culture began (not shown). Within the first 2 days, crypt architecture disappeared to form organoid structures composed of a monolayer of epithelial cells and a central lumen. At days 10–12 of culture, two types of structures were observed: colonospheres, which are composed of a monolayer of epithelial cells with a central lumen and colonoids exhibiting budding crypts (Figure 1). The epithelium of colonospheres and colonoids was polarized, with the basolateral side facing outwards (β-catenin labeling) and the apical side oriented toward the lumen (actin labeling) (Figure 1B).

**FIGURE 2:**
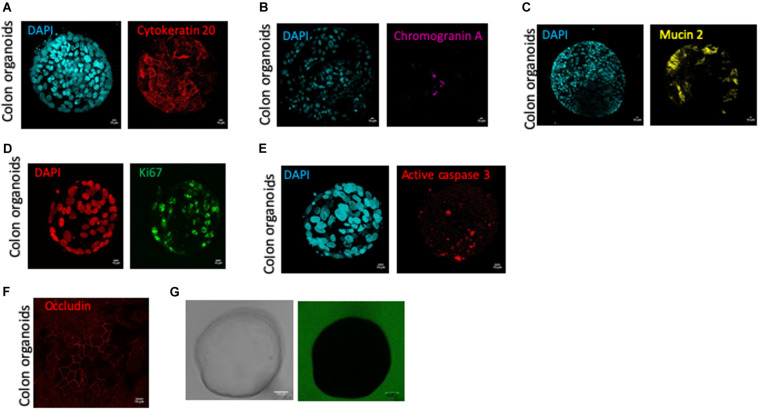
Human colon organoid cell type composition and organoid sealing observation. Colonosphere cross sections are shown for each staining, and all images were acquired with SP8 confocal microscope at 63X objectives and analyzed by Image J software, after 10 days of culture of control organoids. Scale bar 10 μm **(A–G)**. **(A)** DAPI nucleus (cyan) and cytokeratin 20 (red) staining for colonocytes, **(B)** DAPI nucleus (cyan) and chromogranin A (pink) staining for enteroendocrine cells, **(C)** DAPI nucleus (cyan), mucin 2 (yellow) staining for goblet cells, **(D)** DAPI nucleus (red) and ki67 (green) staining for proliferative cells, **(E)** DAPI nucleus (cyan) and active caspase 3 (red) staining for apoptotic cells, **(F)** Occludin staining (red) for tight junction labeling **(G)** One colonosphere cultivated in the presence of FITC-4 KDa (green) in bright field (left) or fluorescence (right) microscopy.

At 10–12-days of culture, human colon organoids had positive staining for cytokeratin 20 (Figure 2A), chromogranin A (Figure 2B), and mucin 2 (Figure 2C), demonstrating that differentiated cells: colonocytes, enteroendocrine cells, and goblet cells respectively, were present in cultured colon organoids (Figure 2). In 12-days cultured organoids, epithelial turnover was present and characterized by Ki67 staining in proliferative zones and active caspase-3 staining in apoptotic zones (Figures 2D,E). Interestingly, apoptosis as revealed by caspase-3 staining was predominantly observed inside the lumen of organoids. Finally, 10–12-days cultured human colon organoids demonstrated functional barrier properties, as observed by cobblestone organization of epithelial cells highlighted by occludin staining (Figure 2F) and by the lack of passage of fluorescent dextran 4-kDa added to the medium (Figure 2G).

### Organoids From IBD Patients Have a Morphological Phenotype Different From Healthy Controls and Retain Some Inflammatory Features

**FIGURE 3:**
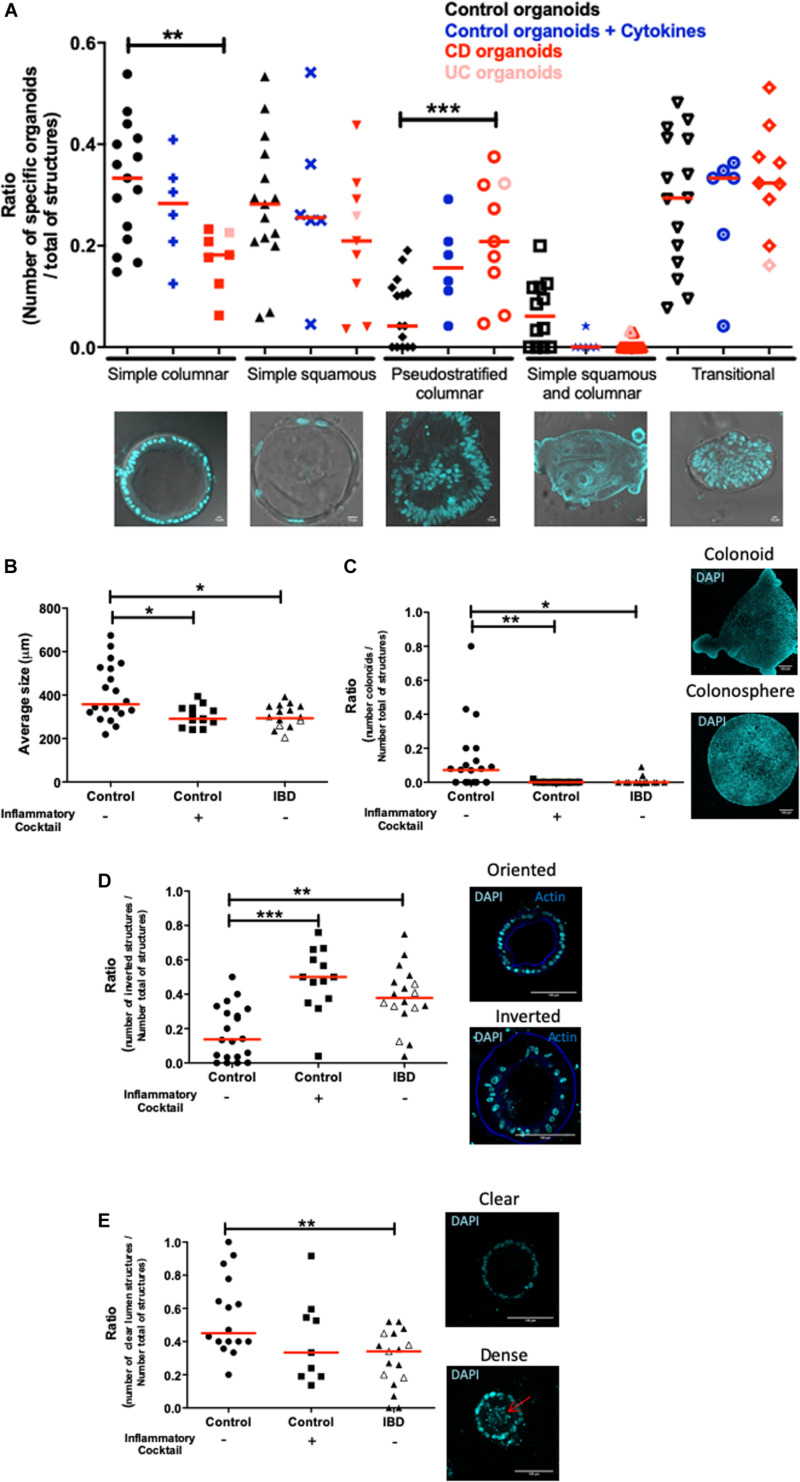
Morphological differences between control and inflamed colon organoids. All images were acquired with SP8 confocal microscope at 20X and 63X objectives and analyzed by Image J software. Red lines on graphs represented medians. *P*-value < 0.05 for *, <0.01 for **, and <0.005 for *** (One-way Anova, (Kruskal Wallis, and Dunns). **(A)** Morphological classification of cultured organoids in subgroups: simple columnar, simple squamous, pseudostratified columnar, simple squamous and columnar, and transitional. Control organoids derived from non-IBD patients were represented by black signs, blue signs represented control organoids treated with an inflammatory cocktail (IL-1β, TNF-α, and IL-6, at 10 ng/ml) from day 3 to day 10. Organoids derived from IBD patient tissues were represented in red: light red for Ulcerative Colitis, dark red for Crohn’s disease. An average of 20 organoids per patient was imaged and analyzed. Medians were represented by red lines. *P*-value < 0.05 (One way anova, Kruskal–Wallis, and Dunns). For **(B–E)**, control organoids cultured from non-IBD patient tissues were represented by circles, square signs represented control organoids treated with an inflammatory cocktail (IL-1β, TNF-α, and IL-6, at 10 ng/ml) from day 3 to day 10, and organoids derived from IBD patient tissues were represented by triangles: open symbols for Ulcerative Colitis and closed symbols for Crohn’s disease. **(B)** Average size of organoids (μm). **(C)** The number of colonoids (budding structures) per total number of organoids (colonospheres plus colonoids each represented on right panel). **(D)** DAPI nucleus (cyan) and actin (blue) labeling were used in cross sections of organoid cultures (right panels) to visualize oriented (actin labeling toward the lumen) (right upper panel) and inverted organoids (actin labeling outwards the lumen) (right bottom panel) (scale bar 100 μm). On the left panel, the number of inverted organoids per total number of organoids (oriented plus inverted) was represented. **(E)** Nuclei labeling in cross sections of organoids to visualize clear (right upper panel) and dense (right bottom panel) lumen, the arrow showed the presence of debris in the lumen. The number of organoids with “clear” empty lumen per total number of organoids (empty, dense and without lumen) was represented.)

Next, crypts isolated from tissues of both non-IBD and IBD patients (Crohn’s and ulcerative colitis) were cultured. In IBD patients, tissues for crypt isolation were harvested at the margin of the inflamed zone, where the epithelium was still intact. Five categories of organoid structures were present in these cultures (Figure 3A). (1) Organoids with a simple columnar epithelium, (2) Organoids with a simple squamous epithelium, (3) Pseudostratified epithelium (4) Organoids with both squamous and columnar epithelium depending on the zones within the organoid and (5) Transitional structures, where the epithelium showed no organization. The proportion of simple columnar organoids was significantly decreased in IBD patient organoid cultures compared to controls (Figure 3A). In contrast, the proportion of pseudostratified structures was significantly increased in IBD patient organoid cultures compared to controls (Figure 3A). This last observation is consistent with the fact that in tissues from IBD patients, pseudostratified epithelium is often observed in healing zones (Feakins and British Society of Gastroenterology., 2013).

In addition, organoids from IBD patients had a significant smaller size than control organoids (Figure 3B). Similarly, the ratio of the number of colonoids (as opposed to colonospheres) over the total number of structures was significantly lower in organoids from IBD patients, as compared to control organoids (Figure 3C). As a matter of fact, mostly immature colonospheres were observed in cultures of organoids from IBD patients. As described above, in organoid cultures from control tissues, the organoids were polarized, with an apical side oriented toward the lumen. We called these organoids “orientated” (see Figure 3D). However, we noticed that in organoid cultures from IBD patients, a large number of organoids had an inverse orientation, with the cortical actin staining (representative of the apical side) facing outwards of the structures (photomicrograph in Figure 3D). We called these structures “inverted” and we quantified them. The proportion of inverted structures in organoid cultures from IBD patient was significantly higher than in cultures of control organoids, with nearly three times more inverted organoids (Figure 3D). Some organoids had debris inside their lumen, most likely due to excessive cell death, while others had clear lumen (Figure 3E microphotographs). We quantified the proportion of “clear” lumen structures and found it significantly lower in organoid cultures from IBD patients compared to controls (Figure 3E).

**FIGURE 4:**
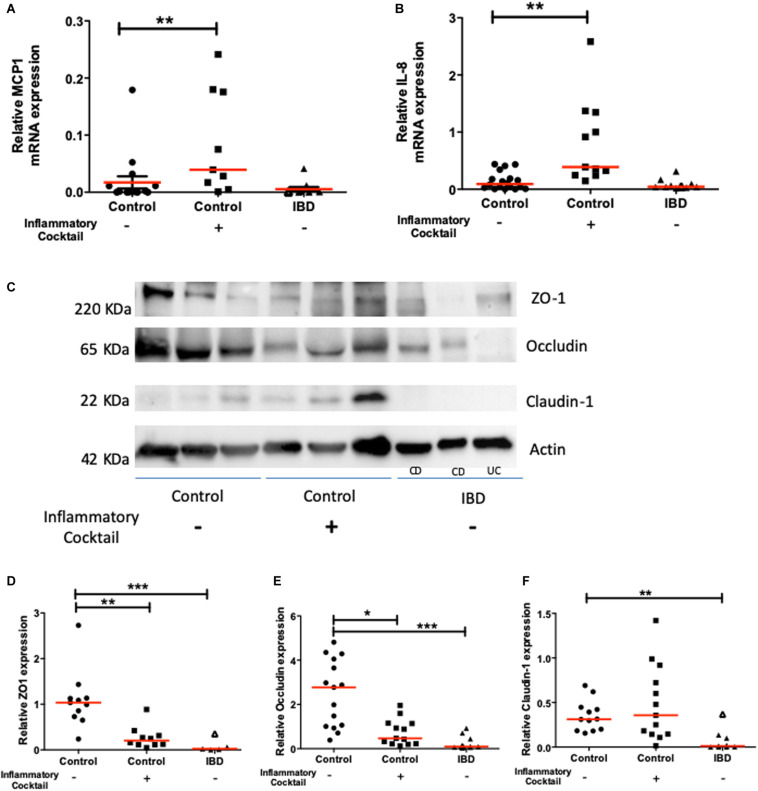
Chemokine (MCP-1 and CXCL-8) and tight junction molecule (ZO-1, Occludin and Claudin-1) mRNA and protein expression respectively, in controls (circles), controls exposed to an inflammatory cocktail (IL-1β, TNF-α, and IL-6, at 10 ng/ml) from day 3 to day 10 (squares) and organoids from IBD patients (triangles): ulcerative colitis (UC) patients (opened triangles) and Crohn’s disease (CD) patients (closed triangles). mRNA or proteins were extracted from organoids after 10 days of culture and data were normalized by GAPDH or actin expression respectively. Red lines represented medians. *P*-value < 0.05 for *, <0.01 for **, and <0.005 for ***(One-way Anova, Kruskal Wallis, and Dunns). **(A)** MCP1 mRNA expression. **(B)** IL-8 mRNA expression. **(C)** Representative Western-blot of the ZO-1, Occludin, Claudin-1 and Actin protein expression in tissues from 3 representative samples from controls (non-IBD), and IBD (Crohn’s disease: CD, and ulcerative colitis: UC) patients. **(D)** Quantification of ZO-1 relative protein expression. **(E)** Quantification of Occludin relative protein expression. **(F)** Quantification of Claudin relative protein expression.

The expression of functional molecules characteristic of an inflammatory phenotype was studied in organoid cultures from IBD patients and controls. First, the mRNA expression of inflammatory cytokines such as MCP1 (Figure 4A), CXCL-8 (Figure 4B), and IP-10 (not shown) was similar in colon organoids from IBD patients or controls. Second, Western blot analysis of tight junction proteins revealed a significant decreased expression of ZO1, Occludin and Claudin-1 for colon organoids from IBD patients compared to controls (Figures 4C–F).

**FIGURE 5:**
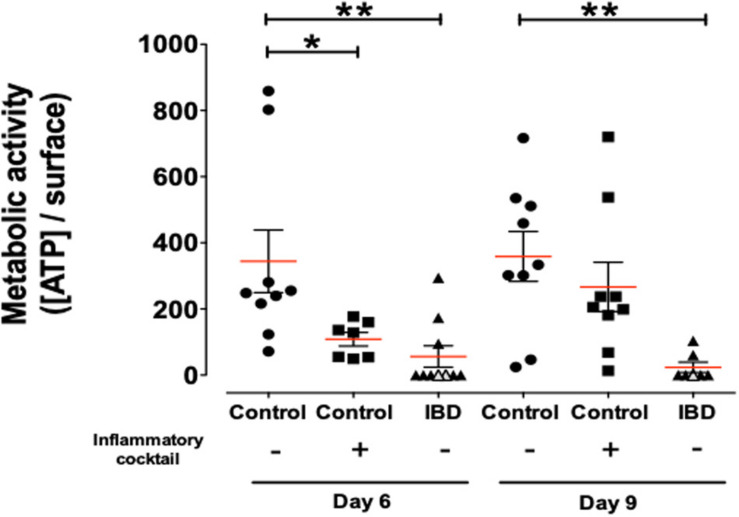
Concentration of ATP release per surface of total organoids as a measure of metabolic activity/cell survival in organoids cultured for 6 and 9-days and harvested from control (non-IBD) patients (circles), control (non-IBD) organoids exposed to inflammatory cocktail (IL-1β, TNF-α, and IL-6, at 10 ng/ml) from day 3 to day 10 (squares) and organoids from IBD patients (triangles): ulcerative colitis (UC) patients (opened triangles) and Crohn’s disease (CD) patients (closed triangles). Red lines represented medians. *P*-value < 0.05 for * and <0.01 for ** (One-way Anova, Kruskal Wallis, and Dunns).

Metabolic status of organoids was investigated by dosing ATP in organoid cultures (Figure 5). Organoids from IBD patients demonstrated a significantly reduced viability/metabolic activity status, compared to cultured organoids from control non-IBD. This significant difference was observed both at day 6 and day 9 of culture (Figure 5).

Taken together, these data demonstrated that cultured colon organoids from IBD patients have a different phenotype from non-IBD controls. After 10–12 days of culture, IBD epithelial organoids retained some inflammatory features, such as pseudostratification, slow growth, altered polarization and decreased expression of tight junction proteins. Interestingly, these inflammatory features appeared both in Crohn’s disease and ulcerative colitis organoid cultures.

### Pro-inflammatory Cocktail Can Induce a Pro-inflammatory Phenotype in Control Organoids

We next investigated whether inflammatory stimulation of control non-IBD organoid cultures could induce an inflammatory phenotype in cultured organoids. We stimulated control colon organoid cultures at day 3 and day 6 of cultures, with an inflammatory cocktail composed of Interleukin-1, Tumor Necrosis Factor-alpha (TNFα) and Interleukin-6 (10 ng/ml each) (Supplementary Figure S1). Inflammatory cocktail stimulation did not significantly change the distribution of organoid categories (columnar, squamous, pseudostratified, transitional, etc.) (Figure 3A). However, incubation with the inflammatory cocktail significantly reduced the size of organoids in cultures (Figure 3B), the proportion of colonoids (Figure 3C) and increased the number of “inverted” organoid structures (Figure 3D), while having no effect on the proportion of “clear lumen” structures (Figure 3E). Incubation with the inflammatory cocktail was also able to induce a significant mRNA overexpression by colon organoids of the two inflammatory chemokines MCP-1 (Figure 4A) and CXCL-8 (Figure 4B), but did not change the expression of IP-10 (not shown). Inflammatory stimulation of control non-IBD organoid cultures significantly decreased the expression of tight junction proteins ZO1, and Occludin, but not Claudin-1, compared to control (unstimulated) condition (Figures 4C–F). Finally, cell viability as observed by measurement of ATP production was also significantly reduced in control organoids incubated with the inflammatory cocktail, but this reduction was observed right after the stimulation (day 6), and did not last at day 9 (Figure 5).

### Cell Composition Is Identical Between Controls and Inflamed Organoids

**FIGURE 6:**
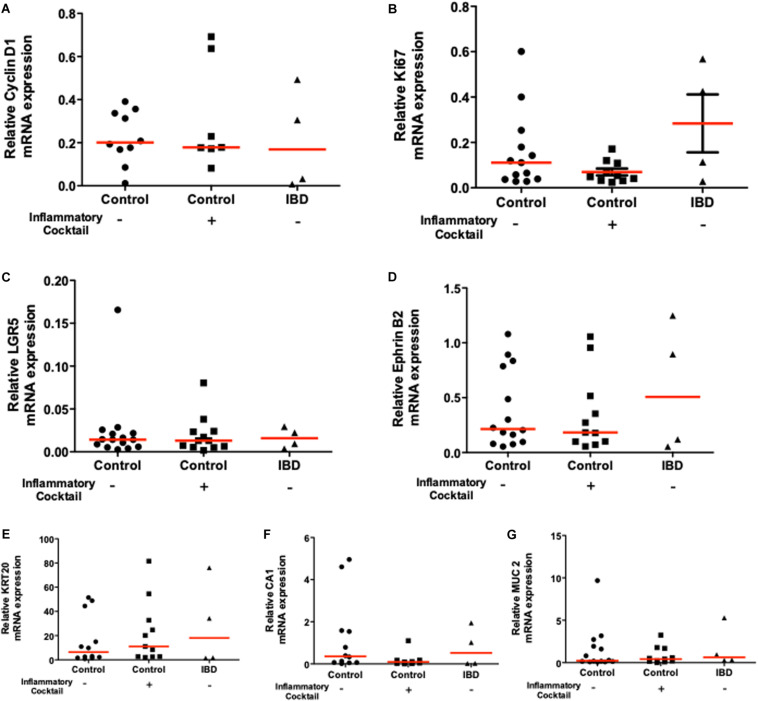
mRNA expression of proliferation markers [Cyclin D1 and Ki67 **(A)** and **(B)** respectively], immaturity markers [LGR5 and Ephrin B2 **(C)** and **(D)** respectively], and differentiation markers [Carbonic Anhydrase-1: CA1, Cytokeratine-20: KRT20 **(E)**, Carbonic Anhydrase-1: CA1 **(F)**, and Mucin 2: MUC2 **(G)**] in 10 days cultured organoids from controls (circles), control organoids exposed to inflammatory cocktail (IL-1β, TNF-α, and IL-6, at 10 ng/ml) from day 3 to day 10 of cultures (squares) and organoids from IBD patients (triangles): ulcerative colitis (UC) patients (opened triangles) and Crohn’s disease (CD) patients (closed triangles). Data were normalized to GAPDH. Red lines in graphs represented medians. No statistical significant difference was observed.

We wanted to determine whether the inflammatory phenotype observed for IBD organoids and for inflammatory cocktail-stimulated organoids could be explained by different organoid cell composition compared to controls. We therefore investigated the mRNA expression of proliferation genes (cyclin D1 and Ki67), the expression of intestinal stem cell markers (LGR5 and Ephrin B2), and the expression of differentiation markers (KRT20, Chromogranin A and MUC2). Cyclin D1 and Ki67, two proliferation markers were similarly expressed in control organoid cultures and in inflamed organoids (both inflammatory cocktail-stimulated and IBD organoids (Figures 6A,B). Intestinal stem cell markers LGR5 and Ephrin B2 were also similarly expressed in control organoids and inflamed organoids (Figures 6C,D). Finally, the differentiation markers KRT20, Chromogranin A and MUC2 were also similarly expressed in the three types of organoid cultures: controls non-IBD, controls non-IBD plus inflammatory cocktail, or IBD organoids (Figures 6E–G).

Taken together, these results suggested that inflamed organoids have similar proliferation, stem cell and differentiation capacities compared to controls. Differences in biological activities of inflamed organoids rather than their cell composition might explain their morphological and functional differences.

### Effects of Clinically Used Treatments on the Inflammatory Phenotype of Human Colon Organoids From IBD Patients

**FIGURE 7:**
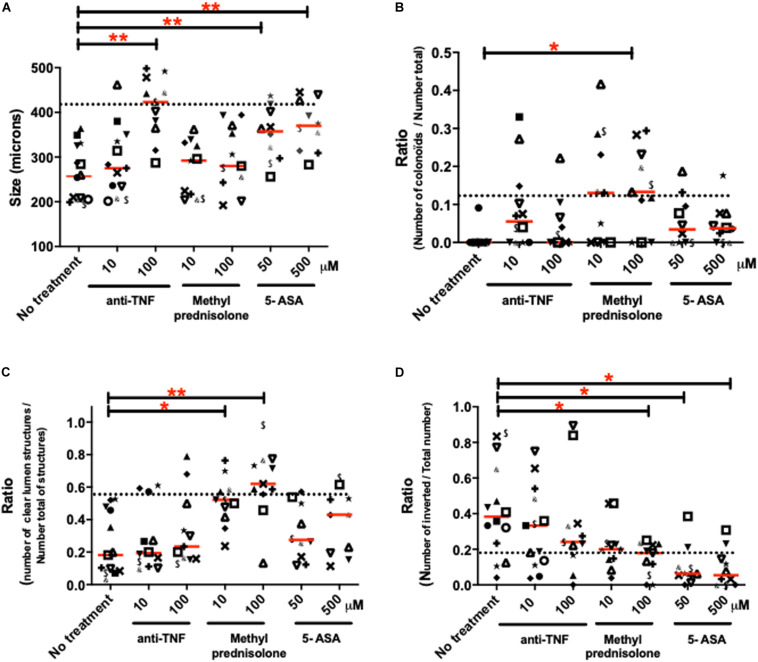
Effects of different treatments used in clinics on the morphological characteristics of IBD organoids. IBD organoids were derived from IBD patients. Crohn’s disease (CD) patient organoids were represented with closed symbols and keyboard symbols, while ulcerative colitis (UC) patient organoids were represented by opened symbols. One patient was represented by the same symbol in all figure panels. IBD organoids were treated from day 0 to day 10 with anti-TNF (Remicade) at 1, 10, and 100 μM, or methyl-prednisolone (Solupred) at 10 and 100 μM, or 5-ASA (Pentasa) at 50 and 500 μM. Where possible, an average of 20 organoids was studied per condition per patient. Medians were represented by red lines on graphs. Doted lines represented for each criteria, the median measured for this criteria in control (non-IBD), non-treated organoid cultures. Statistics were not performed on these graphs due to the low n number in some groups. **(A)** Average size of organoids (μm) **(B)** Number of colonoids (budding structures) per total number of organoids (colonospheres plus colonoïds) **(C)** Number of organoids with empty lumen per total number of organoids (empty, dense and without lumen). **(D)** Number of inverted organoids per total number of organoids (oriented plus inverted).

Human colon organoids from IBD patients were cultured in the presence or not of treatments currently used in the clinics: 5-ASA, methyl-prednisolone and anti-TNF (see Supplementary Figure S2 for protocol). Four morphological parameters significantly altered in IBD organoid cultures compared to controls were followed: (1) the size of organoids (Figure 7A), (2) the proportion of colonoids (budding structures) (Figure 7B), (3) the proportion of structures with empty (clear) lumen (Figure 7C) and (4) the polarity of organoids (Figure 7D). The purpose of these experiments was to investigate whether clinically used treatments can modify the inflammatory phenotype of individual patient organoid cultures. Overall, considering the medians (shown by the line in each groups of Figure 7), all treatments had positive effects on some of the observed parameter, in a dose-dependent manner. However, not all treatments were efficient on all parameters.

Considering the lower size of IBD organoid structures compared to non-inflamed organoids, anti-TNF and 5-ASA, but not methyl prednisolone treatments had a significant effect, increasing the size of organoids compared to the no-treatment condition (Figure 7A). Both anti-TNF and 5-ASA treatments were able to bring the size of some IBD organoid cultures toward the level of controls (doted line) (Figure 7A). Only the highest dose of anti-TNF treatment and the two doses of 5-ASA treatment significantly increased the size of organoids compared to untreated cultures (Figure 7A). The positive effects of 5-ASA and anti-TNF treatments on the size of IBD organoids were observed both on Crohn’s and ulcerative colitis organoid cultures (Figure 7A).

IBD organoid cultures showed a very low proportion of budding structures compared to controls. This is characterized by low counts of colonoids (Figures 3C, 7B). All three treatments (anti-TNF, methyl prednisolone and 5-ASA) trended to bring the proportion of colonoids to control levels (doted line), but only the highest dose of prednisolone treatment was able to increase significantly the proportion of budding structures, compared to the untreated culture condition (Figure 7B). Positive effects of treatments on colonoid counts were observed both in Crohn’s disease and ulcerative colitis organoid cultures (Figure 7B).

The number of empty (clear) lumen structures in organoid cultures was significantly decreased in IBD organoids compared to non-inflamed organoids (Figure 3E). This decreased proportion of organoids with clear lumen was unchanged in cultures containing anti-TNF or 5-ASA, but was significantly increased by the two doses of prednisolone added to the culture media (Figure 7C). For this parameter as well, the positive effect of prednisolone treatment was observed similarly in Crohn’s disease and ulcerative colitis organoid cultures (Figure 7C).

Finally, it was observed that organoids from IBD patients had an increased inverted polarization compared to non-inflamed controls (Figures 3D, 7D). Treatment with anti-TNF did not reduce significantly the proportion of inverted organoids compared to untreated conditions, but the median of highest dose of anti-TNF treatment was close to the uninflamed (non-IBD) level (doted line). The highest dose of ethyl prednisolone treatment and the two doses of 5-ASA treatments significantly reduced the number of inverted organoids compared to untreated culture (Figure 7D). The positive effect of treatment was observed similarly in Crohn’s disease and ulcerative colitis organoid cultures (Figure 7D).

## Discussion

The present study brings a number of new and important information on organoid cultures. It characterizes both morphological, and gene expression changes in primary organoid cultures from IBD patients, compared to primary organoid cultures from the colon of non-IBD individuals. Importantly, these data demonstrated that even after 10–12 days of culture, and in absence of any further immunological stimulation (no CD45 staining in isolated crypts), crypt stem cells isolated from IBD tissues regrow abnormal epithelium. This could be due to the long-term impregnation to inflammatory mediators of crypts isolated from IBD patient tissues. To grow organoids, tissues were harvested at the margin of inflamed tissues in non-inflamed zones. This choice was made in order to be certain that the isolated epithelium would be intact of ulcers and that sufficient and reproducible crypt numbers would be isolated. Therefore, isolated crypts might have been exposed chronically to adjacent inflammatory mediators. Such chronic exposure of intestinal epithelium to inflammatory mediators could induce epigenetic changes that are conserved in organoid cultures even in the absence of inflammatory mediators. Indeed, epigenetic variations have been identified in tissues from IBD patients (Kellermayer, 2012; McGovern et al., 2015). Interestingly, another recent study performed with organoid cultures from ulcerative colitis patients also suggests an imprinting of IBD stem cells (Dotti et al., 2017). In that study, authors identified a set of genes that exhibited sustained differential expression in cultured organoids from ulcerative colitis patients. These genes include LYZ, CLDN18, hZG16, hCLCA1, MUC12, and AQP8, which are involved in antimicrobial, barrier, mucus production or water transport functions. Another study recently confirmed that differential expression of mucus production gene (MUC2) is observed in organoids from IBD patients, and in particular from Crohn’s disease patients, compared to controls (Noben et al., 2017). Taken together, such data implies that long-lasting epithelial defects are present in IBD patient epithelium, and may be acquired during the course of the disease thereby contributing to the perpetuation of the pathology. Another hypothesis to explain the IBD phenotype in organoid cultures, is that some genetic mutations that have been associated with IBD are involved in architectural organization of the colonic epithelium, conferring a permanent epithelial phenotype. This could be confirmed in future studies that would perform IBD organoid cultures after several cell passages, but it was not the purpose of the present study. Large-scale genome-wide association studies have identified more than 200 loci associated with IBD (Liu et al., 2015). Some of these genes are involved in innate immune response, impaired mechanisms of phagocytosis and autophagy, T cell signaling, but also epithelial barrier function (Prager et al., 2015). For instance, genes such as *ECM1, CDH1, LAMB1, ADAM17A, KIND1/FERMT1* or *HNF4*α are known to be involved in barrier function and single nucleotide polymorphism has been associated with IBD for these genes (Kern et al., 2007; Blaydon et al., 2011; Bianco et al., 2015). If such polymorphism is associated with a functional phenotype of impaired barrier function, then this could explain the decreased expression of tight junction proteins we observed in cultured organoids. The *OSMR* gene coding for the Oncostatin M receptor is also associated with IBD and is known to promote epithelial cell proliferation. Here again, such mutation could explain the smaller size of IBD organoids we observed (Beigel et al., 2014; Bianco et al., 2015). Mutations in the type VII collagen gene *COL7A1* are associated with IBD (Freeman et al., 2008; Uhlig, 2013). Considering the major role of type VII collagen in keratinocyte polarity (Dayal et al., 2014), a similar role could be proposed in intestinal epithelial cells, although no studies have yet investigated such role in this cell type. The inverted phenotype that we observed in 70% of IBD organoids could be explained by such mutation. Future studies will be needed to investigate the association of IBD gene polymorphism with specific organoid culture phenotype. However, our present study also highlights the possibility for pharmacological intervention to revert, at least in part, some of the inflammatory phenotype of organoid cultures. This could undermine the importance of genetic defects in IBD-associated epithelial dysfunctions. Altogether, the present model of primary organoid cultures could be considered as a most relevant model to recapitulate *in vitro*, the epithelial defects associated with IBD, and to potentially follow IBD epithelial features.

Our results generated with IBD patient organoid cultures suggested that inflamed organoids have similar proliferation, stem cell and differentiation capacities compared to controls. This was evidenced by the conserved expression of proliferation genes cyclin D1 and Ki67, of intestinal stem cell markers LGR5 and Ephrin B2, and of differentiation markers KRT20, Chromogranin A and MUC2. Similar results were reported by a recent study in which, organoid cultures from ulcerative colitis patients were compared to organoid cultures from non-inflamed controls, showing similar self-renewal capacity and comparable molecular features (Dotti et al., 2016). Importantly, the organoid structures that were followed in the present study expressed the cytokeratin-20 (KRT20) protein (Figure 2A), providing proofs of success in achieving human primary keratinocyte cultures as previously demonstrated (Torreggiani et al., 2019), and confirming the epithelial origin of those cells.

A second important point highlighted by the present study is that the phenotype of inflamed organoid can be recapitulated *in vitro*, by exposing control organoids to an inflammatory cocktail. We showed that a cytokine cocktail induced in control organoid cultures, the same size reduction, inverted phenotype, decreased proportion of colonoids, ZO-1 and Occludin decreased expression, decreased metabolic activity as it was observed in IBD patient organoid cultures. This suggests that *in vivo* as well, these parameters might be under the long-term control of inflammatory mediators. However, some other features of IBD organoids were not recapitulated in control organoids exposed to the inflammatory cocktail. This involves the increased presence of apoptotic debris in the lumen of IBD organoids, and their decreased expression of Claudin-1. This means that for these parameters, short-term exposure to inflammatory mediators might not be sufficient to induce this particular phenotype. Rather, genomic imprinting or mutation might be involved to induce these epithelial phenotypes. Indeed, a frequent occurrence of private variants of the X-linked inhibitor of apoptosis protein has been associated with Crohn’s disease (Zeissig et al., 2015). Importantly, some inflammatory features such as chemokine (CXCL-8 and MCP-1) release are induced by transient exposure to inflammatory cocktail, but are not maintained in IBD organoid cultures (Figures 4A,B). This could mean that in the context of IBD, the regulation of those genes does not depend on long-term imprinting or gene mutation, but is a consequence of acute inflammatory response. Therefore, this model of stimulated organoid culture appears as a unique new tool that will help defining intestinal epithelium gene regulation by individual or combined mediators. In addition, it will clearly serve to screen the efficacy of new therapeutic approaches targeting the effects of inflammatory mediators on epithelial repair.

A third major point raised by the present study is the demonstration that IBD organoid cultures could be used to test the effects of therapeutic options on epithelial biology. We demonstrated that overall, all treatments (anti-TNF, corticosteroids and 5-ASA) had positive impact on the observed parameters in a dose-dependent manner. This is important as it means that such treatments could participate at least to some extent, to epithelial regeneration and repair. The fact that these treatments positively impacted inflamed IBD organoid cultures seems surprising as these treatments were originally developed to target the immune response. It was interesting to note that not all treatments were efficient on the same parameters, and also that some treatments could be more efficient than others in some patients. This first result paves the way to a more extensive study, including large numbers of patients that could aim at defining whether the response of culture organoids could be predictive of the patient’s response to treatment. Similarly, the effects of new therapeutic options focusing on epithelial repair could therefore be tested directly in tissues from patients, and their efficiency could be compared to that of clinically used treatments.

## Conclusion

In conclusion, the extensive characterization of colon organoid cultures from IBD patients compared to non-IBD controls that we have performed in the present study brings important new knowledge. First, it demonstrates the possible use of such model to further characterize epithelial dysfunctions associated with IBD. Second, we showed that this model constitutes a reliable human preclinical model to investigate new therapeutic approaches targeting epithelial repair.

## Data Availability Statement

All datasets generated for this study are included in the article/Supplementary Material, further inquiries can be directed to the corresponding author.

## Ethics Statement

The studies involving human participants were reviewed and approved by either the MICILIP research protocol (National Ethics Committee #NCT01990716), or the biocollection for human samples that included colonic resections (CODECOH national agreement: Colic collection: DC2015-2443). The patients/participants provided their written informed consent to participate in this study.

## Author Contributions

EA, MQ, and MS have performed the experiments, analyzed the data, and contributed to the manuscript drafting. DB, SK, GP, J-PD, SA, CD, LA, and EM have provided technical and/or material support, and contributed to manuscript editing. SC, PL, CD, CR-S, AF, EM, and NV have analyzed and critically reviewed the data, obtained funding, and contributed to the manuscript editing. EA and NV performed the study design, concept, supervision, and manuscript writing.

## Conflict of Interest

SC and PL were employed by the company Urosphere SAS. The remaining authors declare that the research was conducted in the absence of any commercial or financial relationships that could be construed as a potential conflict of interest.

## Supplementary Material

FIGURE S1 Protocol for the culture of organoids from IBD and non-IBD patients. Inflamed organoids were obtained from colon biopsies or resection of IBD patients. But inflamed organoids could be obtained from control patients. When control organoids were formed (at day 3) a chronic inflammatory stress (IL-1β, TNF-α, and IL-6, at 10 ng/ml) was applied at each change of medium. After 10 days of culture morphological and functional study was performed as well as expression of pro-inflammatory mediators or expression of tight junction proteins.

FIGURE S2 Inflamed organoids obtained from colon biopsies or resection of IBD patients were subjected or not to a clinical treatment [anti-TNF (10 or 100 μM); methyl-prednisolone (10 or 100 μM); 5-ASA (50 or 500 μM)]. Treatments were added at day 0 and at each medium change. After 10 days of culture morphological study was performed.

TABLE S1 Characteristics and outcomes of control and IBD patients.

TABLE S2 Antibodies used for immunofluorescence labeling (IF) and western blot (WB) studies.

TABLE S3 Primers used for quantitative RT-PCR studies (from 5′ to 3′).

## Abbreviations

CD: Crohn’s disease
IBD: inflammatory bowel diseases
UC: ulcerative colitis.
