# Supplementary figures and images for: Characterization of Human Colon Organoids From Inflammatory Bowel Disease Patients
Source: Front Cell Dev Biol. 2020 Jun 4;8:363. doi: 10.3389/fcell.2020.00363 (PMC7287042; doi:10.3389/fcell.2020.00363)

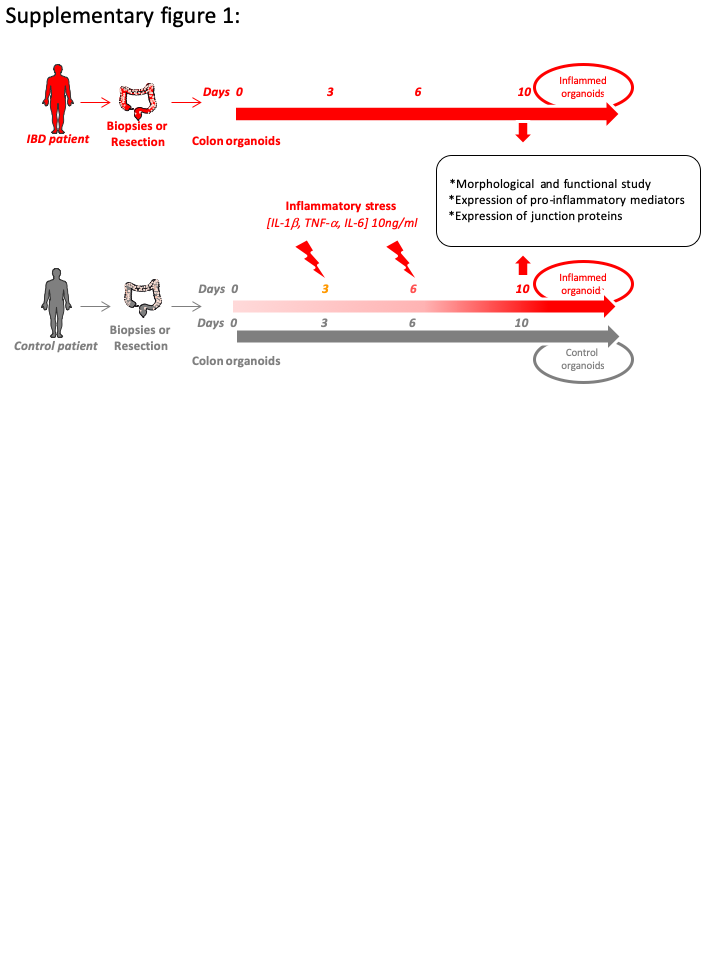

Supplement: FIGURE S1 — Protocol for the culture of organoids from IBD and non-IBD patients. Inflamed organoids were obtained from colon biopsies or resection of IBD patients. But inflamed organoids could be obtained from control patients. When control organoids were formed (at day 3) a chronic inflammatory stress (IL-1β, TNF-α, and IL-6, at 10 ng/ml) was applied at each change of medium. After 10 days of culture morphological and functional study was performed as well as expression of pro-inflammatory mediators or expression of tight junction proteins. [file Image_4.TIFF]

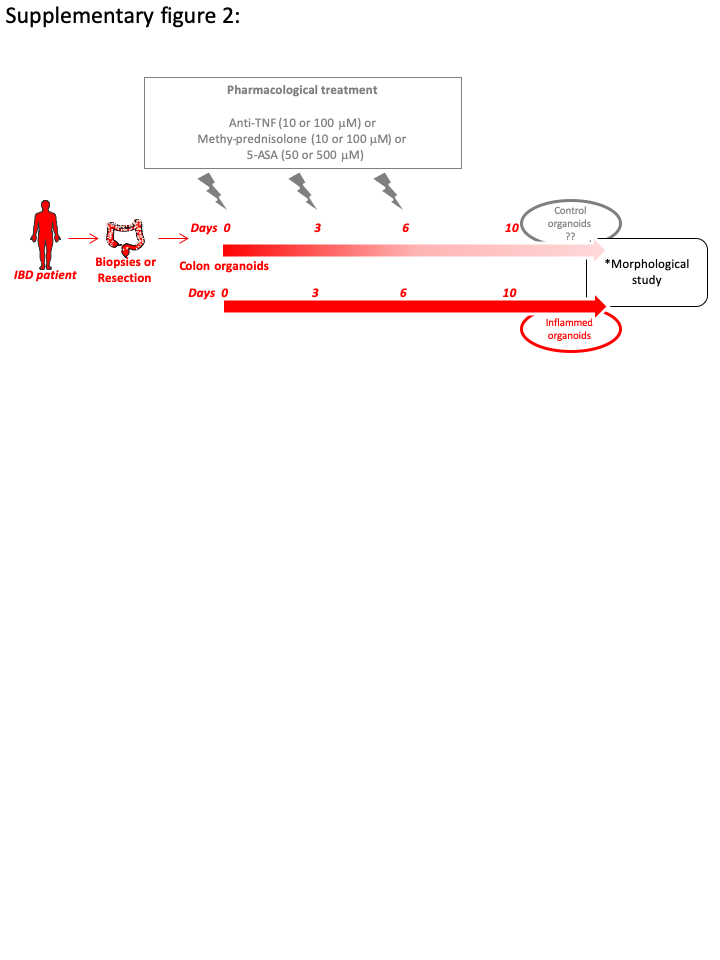

Supplement: FIGURE S2 — Inflamed organoids obtained from colon biopsies or resection of IBD patients were subjected or not to a clinical treatment [anti-TNF (10 or 100 μM); methyl-prednisolone (10 or 100 μM); 5-ASA (50 or 500 μM)]. Treatments were added at day 0 and at each medium change. After 10 days of culture morphological study was performed. [file Image_5.TIFF]

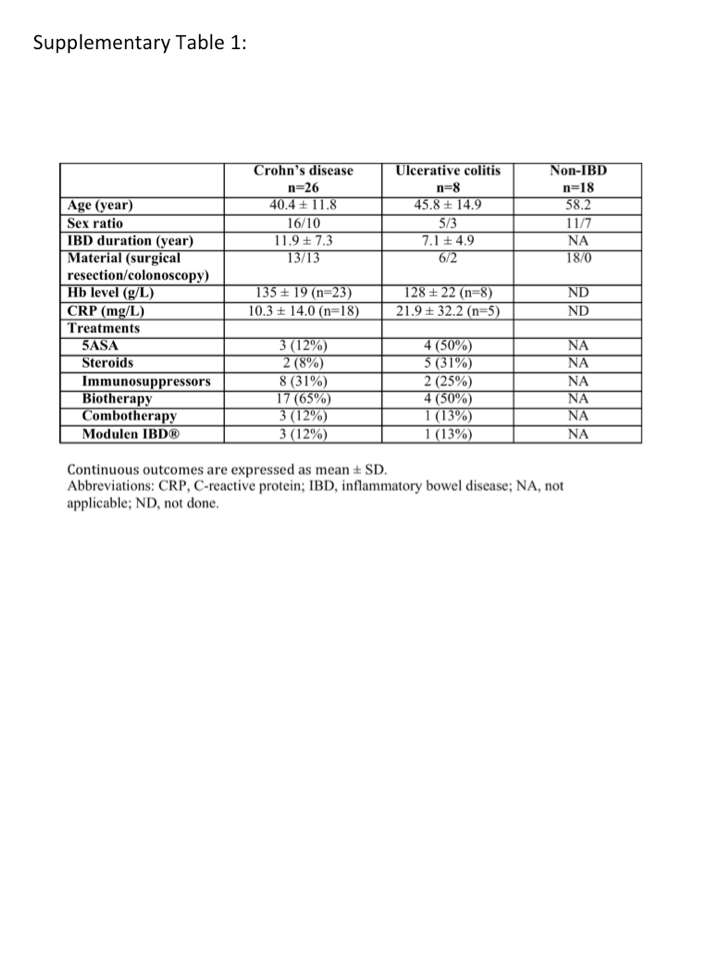

Supplement: TABLE S1 — Characteristics and outcomes of control and IBD patients. [file Image_1.TIFF]

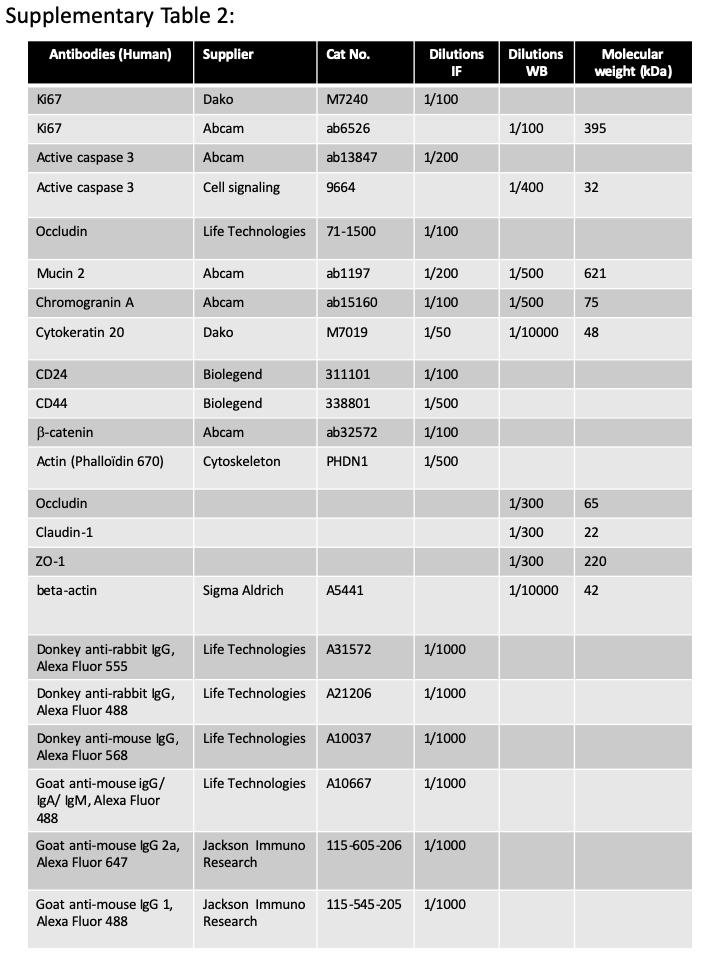

Supplement: TABLE S2 — Antibodies used for immunofluorescence labeling (IF) and western blot (WB) studies. [file Image_2.TIFF]

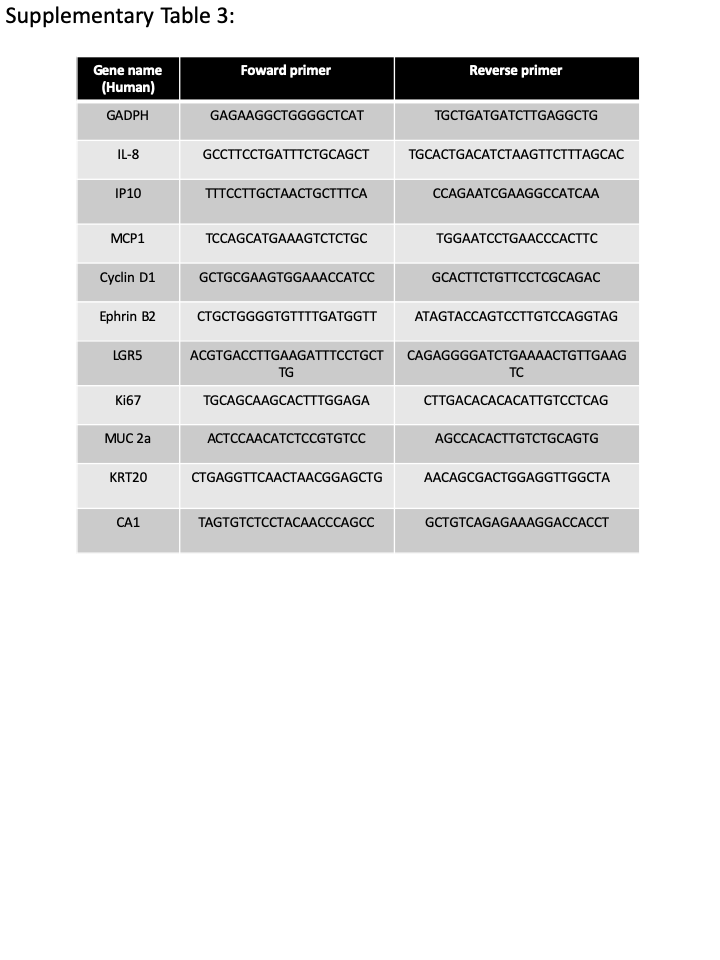

Supplement: TABLE S3 — Primers used for quantitative RT-PCR studies (from 5′ to 3′). [file Image_3.TIFF]
