# Supplementary material for: Characterization of Human Colon Organoids From Inflammatory Bowel Disease Patients
Source: Front Cell Dev Biol. 2020 Jun 4;8:363. doi: 10.3389/fcell.2020.00363 (PMC7287042; doi:10.3389/fcell.2020.00363)
Supplement: Supplementary file 6 [file Data_Sheet_1.PDF]

Supplementary information on Methods

Isolation and culture of colon crypts

Fresh colonic tissues or biopsies were kept for few minutes in Phosphate Buffered Saline solution without calcium and magnesium (PBS WO, Sigma Aldrich) at 4°C. Samples were washed in PBS WO until supernatant was clear. For resection tissues, muscle layer and sub-mucosa were removed and incubated with a mixture of antibiotics (normocin (Invivogen), gentamycin (Sigma Aldrich), and fungizone (Invitrogen)) 4 times for 5 minutes at room temperature (RT). Mucosa was cut into small pieces and incubated 3 times for 5 minutes at RT in 10 mM dithiotreitol (DTT, Roche). Next, samples were transferred in 8 mM ethylenediaminetetraacetic acid (EDTA, Ambion) and slowly rotated for 1 hour at 4°C. After removal of the EDTA, tissue fragments were vigorously re-suspended in PBS to isolate intestinal crypts. Heat inactivated Fetal Bovine Serum (FBS, Life Technologies) was added (5%) and crypts were centrifuged at 40g for 2 minutes. Supernatant was replaced by basal medium (advanced DMEM/F12 (Invitrogen), 2 mM Glutamax (Invitrogen), 10 mM hepes (Gibco) and 5% FBS) and fractions were centrifuged at 500 rpm for 2 minutes. This washing procedure was repeated 3 times, pellet was diluted in 5 ml of basal medium and crypt number was counted. Isolated intestinal crypts (50-100 crypts per 25 µl of Matrigel per well) were embedded in hESC-qualified matrix (Matrigel, Corning) on ice and seeded in 48 wells plates (Greiner) or 8 wells chamber slide (lab-tek II, Nunc). The Matrigel was incubated for polymerization for 10 minutes at 37 °C, and was supplemented with 250 µl/well of complete medium A (advanced DMEM/F12, 2mM Glutamax, 10 mM hepes, 1X N2 (Invitrogen), 1X B27 minus vitamin A (Life technologies), 1X penicillin/streptomycin solution (Invitrogen), 50 ng/ml human EGF (Gibco), 100 ng/ml

human noggin (Tebu), 100 ng/ml human Wnt3a (R&D systems), 1 mM N-acetylcysteine (Sigma Aldrich), 1μg/ml human R-spondin (R&D systems), 10 mM nicotinamide (Sigma Aldrich), 10 nM gastrin (Sigma Aldrich), 10 μM SB202190 (Sigma Aldrich), 0.01 μM PGE_2 (Sigma Aldrich), 0.5 μM LY2157299 (Axon MedChem)). Medium was changed every other day with complete medium B (advanced DMEM/F12, 2mM Glutamax, 10 mM hepes, 1X N2, 1X B27 minus vitamin A, 1X penicillin/streptomycin solution, 50 ng/ml human EGF, 100 ng/ml human noggin, 100 ng/ml human Wnt3a, 0.5 μg/ml human R-spondin, 10 mM nicotinamide, 10 nM gastrin, 10 μM SB202190, 0.01 μM PGE_2) and culture was maintained in a humidified incubator at 37°C under 5% CO_2 and 95% air atmosphere for an average of 10 days.
